# Plant Extracts as Alternative Additives for Sperm Preservation

**DOI:** 10.3390/antiox10050772

**Published:** 2021-05-13

**Authors:** José Luis Ros-Santaella, Eliana Pintus

**Affiliations:** Department of Veterinary Sciences, Faculty of Agrobiology, Food and Natural Resources, Czech University of Life Sciences Prague, Kamýcká 129, 165 00 Prague 6-Suchdol, Czech Republic; pintus@af.czu.cz

**Keywords:** antioxidants, artificial insemination, cryopreservation, herbal pharmacognosy, oxidative stress, phytotherapy, plant extract, semen storage, spermatozoa

## Abstract

Sperm preservation is a crucial factor for the success of assisted reproductive technology (ART) in humans, livestock, and wildlife. Irrespective of the extender and the storage conditions used, semen handling and preservation negatively affect sperm quality. Moreover, oxidative stress, which often arises during semen storage, significantly reduces sperm function and compromises the sperm fertilizing ability by inducing oxidative damage to proteins, lipids, and nucleic acids. Plant extracts have recently emerged as a cheap and natural source of additives to preserve and enhance sperm function during semen storage. The present work provides an update on the use of these natural compounds as alternative additives for sperm preservation in 13 animal species, including humans. A detailed description of the effects of 45 plant species, belonging to 28 families, on sperm function during semen storage is presented. The plant material and extraction method employed, dosage, possible toxic effects, and antimicrobial properties are provided.

## 1. Introduction

The use of assisted reproductive technology (ART) is nowadays a common praxis applied to livestock breeding, wildlife conservation, and human fertility treatment. Artificial insemination (AI), which entails the collection and processing of the male gamete, is the ART most popularly applied to domestic animals thanks to the several advantages that it provides to the breeders compared to natural mating. For instance, AI favors the interchange of genetic lines and, the selection of the best-quality sires by the creation of germplasm banks, while it helps to considerably reduce the spread of disease and increases the animals’ reproductive performance. Furthermore, due to the accelerated biodiversity loss, ARTs are of paramount interest in conservation breeding programs (CBPs). Within the most threatened vertebrate taxa are amphibians and freshwater fishes. Thus, for instance, the use of cryopreserved sperm in amphibians from CBPs allows the fertilization of a single female’s spawn with spermatozoa from many genetically diverse males without the need of animal transport [1]. In humans, the employment of ARTs is increasing considerably due to the significant growth in infertility cases in the last few decades. Moreover, semen cryopreservation is the most suitable option to preserve the fertility of men undergoing cancer treatment [2]. In this sense, it is important to optimize the protocols for semen storage by increasing sperm life span without compromising sperm function. This would translate into increased fertilization rates in AI outcomes. Plant extracts have recently emerged as a cheap and natural source of additives to preserve and enhance sperm function during semen storage. Most plant species are considered powerful sources of antioxidants, which can act as reactive oxygen species (ROS) scavengers for palliating the deleterious effects of oxidative stress on sperm function. Moreover, these natural compounds can have antimicrobial properties and increase the activity of several antioxidant enzymes. The present work aims to provide an overview of the use of plant extracts as natural compounds for sperm preservation in different animal species (including humans) and their effects on sperm function. Extraction methods, suitable concentrations to be used for the different species evaluated, and the possible side effects of the plant extracts are also provided.

## 2. Semen Preservation and Its Effects on Sperm Quality

Artificial insemination is usually carried out with semen stored in a liquid or frozen state. The cryopreserved semen is commonly employed in the AI of domestic and wild animals. However, in species such as pigs, goats, and horses, the most common way to preserve the sperm cells is by refrigeration at 5–17 °C for several days. In general, there are two types of semen extenders for sperm liquid storage: short- and long-term extenders. The first one is intended for semen preservation for up to three days, while the long-term type is able to preserve the sperm cells for more than three days thanks to the supplementation with additives, which significantly increase the price of the extender. The composition of semen extenders varies considerably depending on the species and type of sperm preservation (i.e., cryopreserved or refrigerated). In general, the semen extender contains nutrients (mainly monosaccharides), cryoprotectant agents (CPAs) as protection against cold shock, buffers, antioxidants, and antibiotics [3]. The CPAs are mainly employed for sperm cryopreservation but also for sperm storage at temperatures ≤5 °C. There are two types of CPAs: permeable and non-permeable. Among the most popular permeable CPAs are glycerol, dimethyl sulfoxide (DMSO), and ethylene glycol (EG), whereas for non-permeable CPAs, proteins found in egg yolk and milk as well as sugars are preferred [4,5]. Due to the possible overproduction of ROS during semen preservation, the addition of antioxidant compounds to the semen extender is common. For sperm cryopreservation, antioxidants can be added to the extender before freezing or after semen thawing. With the use of antioxidants, the amount of ROS produced by semen handling and the cryopreservation process is considerably reduced. Among the most common antioxidants employed for sperm freezing are vitamin E (α-tocopherol/Trolox), glutathione (GSH), SOD, catalase (CAT), vitamin C (ascorbic acid), quercetin, and melatonin [6,7]. In boar, for example, bacterial contamination is frequently detected both in raw and diluted semen as the addition of antibiotics (single or combination) is a common praxis during semen preservation [8]. As alternatives to these compounds, natural additives such as plant extracts can provide protection against both ROS attack and microbial contamination.

Irrespective of the extender and the storage conditions used, semen handling and preservation negatively affect sperm quality. Despite being a necessary step to increase sperm lifespan and the number of AI doses, semen dilution decreases the levels of antioxidant enzymes found in the seminal plasma, thus reducing their protection against oxidative stress. Seminal plasma contains several antioxidant enzymes; superoxide dismutase (SOD) and glutathione peroxidase (GPx) are those with the greatest activity [9]. Additionally, semen samples stored at 5 °C usually contain egg yolk, which, on one hand, protects against cold shock and, on the other hand, promotes the formation of hydrogen peroxide (H_2_O_2_) [10,11]. Oxidative stress, which often arises during semen storage, significantly reduces sperm function and compromises the sperm fertilizing ability by inducing oxidative damage to proteins, lipids, and nucleic acids [12]. Another factor that causes a decline in sperm parameters during semen storage is bacterial contamination, which appears prevalent in extended semen given the resistance of many microorganisms to the most commonly used antibiotics [13,14].

The semen cryopreservation process alters the cellular and molecular structure of spermatozoa. Several factors, such as semen handling, cold shock, osmotic and toxic stresses induced by cryoprotectants, and the formation/reshaping of extra- and intracellular ice during freezing and thawing, contribute to the poor sperm quality observed after semen thawing. All of these factors promote a decrease in sperm motility, viability, and mitochondrial membrane potential together with plasma membrane disruption, volumetric changes, and sperm flagellum alterations [15,16,17]. Approximately 40–50% of spermatozoa do not withstand the cryopreservation process even with optimized protocols [18]. Moreover, during the freezing and thawing process, the proteome, transcriptome, and epigenome are also modulated [19]. Recently, it has been reported that the cryopreservation process activates the autophagy pathway by inducing an increase in the conversion of LC3-I to LC3-II protein, which is associated with a decrease in sperm viability [20]. Thus, the cryopreservation procedure affects the expression of genes and proteins related to sperm fertility, motility, viability, acrosome integrity, and mitochondrial activity, among others.

## 3. Herbal Pharmacognosy and Its Use in Andrology

Pharmacognosy is defined as the study of drugs from natural sources such as microorganisms, plants, fungi, and animals. Thus, herbal pharmacognosy (reviewed in [21]) is focused on the study of plants and their derived compounds as possible therapeutic agents. For thousands of years, plants have been used as drugs to treat numerous illnesses (wounds, burns, and insect bites), being the basis of many modern medicines and a source of new drugs in pharmacology. Plants produce a wide range of chemical compounds (phytochemicals) that protect them against potential pathogens (bacteria, fungi, viruses), herbivores, and pests as well as attracting pollinators and facilitating symbiosis with other organisms. These phytochemicals are known as secondary metabolites and are derived from primary metabolites (involved in normal growth, development, and reproduction) such as carbohydrates, lipids, and amino acids, among others. Secondary metabolites have been widely used by humans (e.g., pigments, condiments, medicines) and are the main compounds responsible for the pharmacological activity of plants. Many drugs currently used in modern medicine (atropine, codeine, morphine, taxol, etc.) are derived from plant secondary metabolites. The major groups of secondary metabolites found in plants include alkaloids, phenolic compounds, and terpenoids. Numerous valuable properties such as antioxidants, antimicrobial, antiviral, antitumor, analgesic, anti-inflammatory, antimutagenic, and antiparasitic have been linked to these compounds.

The male factor accounts for around 50% of human infertility, which is a multifactorial disorder that often cannot be treated. The etiology of male infertility is unknown in approximately half of cases and is called “idiopathic infertility”. As a cheap and natural remedy to treat some male fertility disorders, herbal medicine has become an effective means of treatment. Oxidative stress is a possible factor that affects many patients with fertility disorders, and numerous plant species have been used as a source of antioxidants, with promising results [22]. Recently, in humans, a positive relationship has been reported between the antioxidant capacity of seminal plasma with sperm concentration and total motility [23]. Furthermore, plants and their derived compounds have been used as diet supplements for the treatment of fertility disorders as well as for the improvement of reproductive performance in livestock, wildlife, and humans [24,25,26].

As previously discussed, semen preservation (especially cryopreservation) promotes damage to sperm structure and thus to sperm function. Moreover, the overproduction of ROS during sperm storage can lead to oxidative stress, compromising the sperm integrity and fertilizing ability. In order to palliate these undesirable side effects of sperm storage, several compounds have been added to the semen extender in order to optimize the preservation protocols. Most of these substances are chemically synthesized and their prices are usually elevated (especially some antioxidants). On the other hand, in the last two decades, the use of plant extracts, as an economic and natural source of antioxidants, has become a new approach for the preservation of semen, with promising results.

### 3.1. Plant Families and Species Used as Preservatives for Sperm Preservation

**Figure 1:**
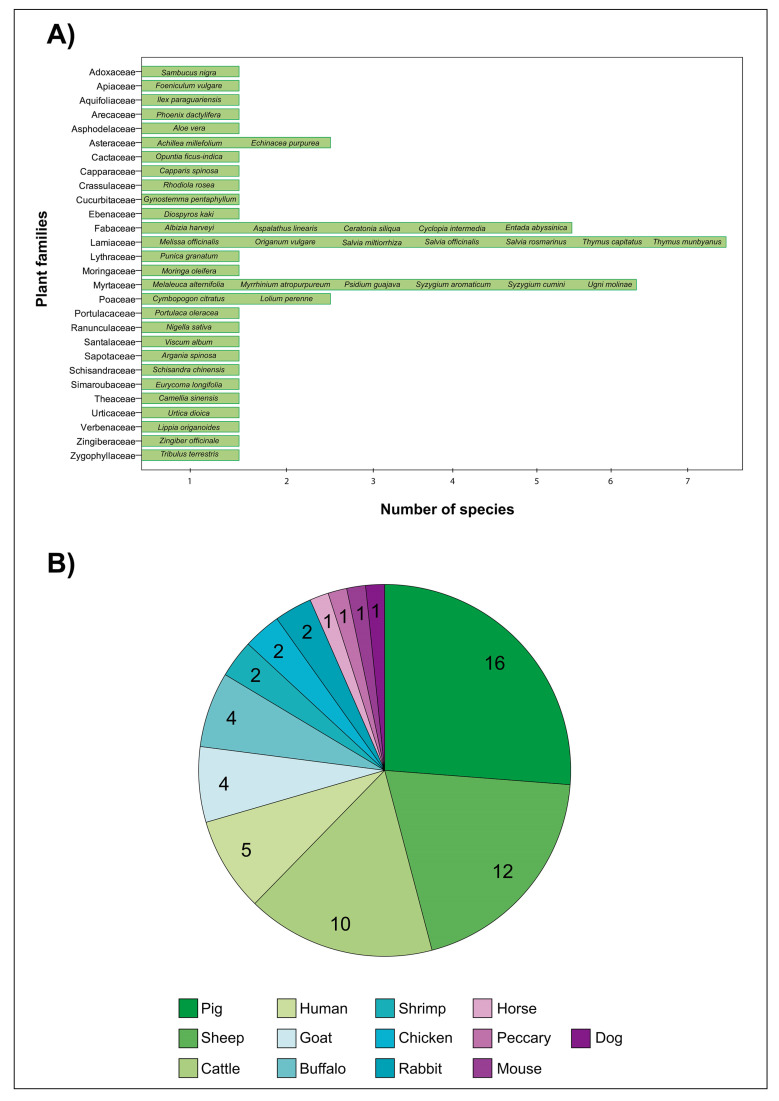
(**A**) Plant families (*N* = 28) and species (*N* = 45) overviewed in the present work. (**B**) Number of plant species tested in the spermatozoa from different animal species.

**Figure 2:**
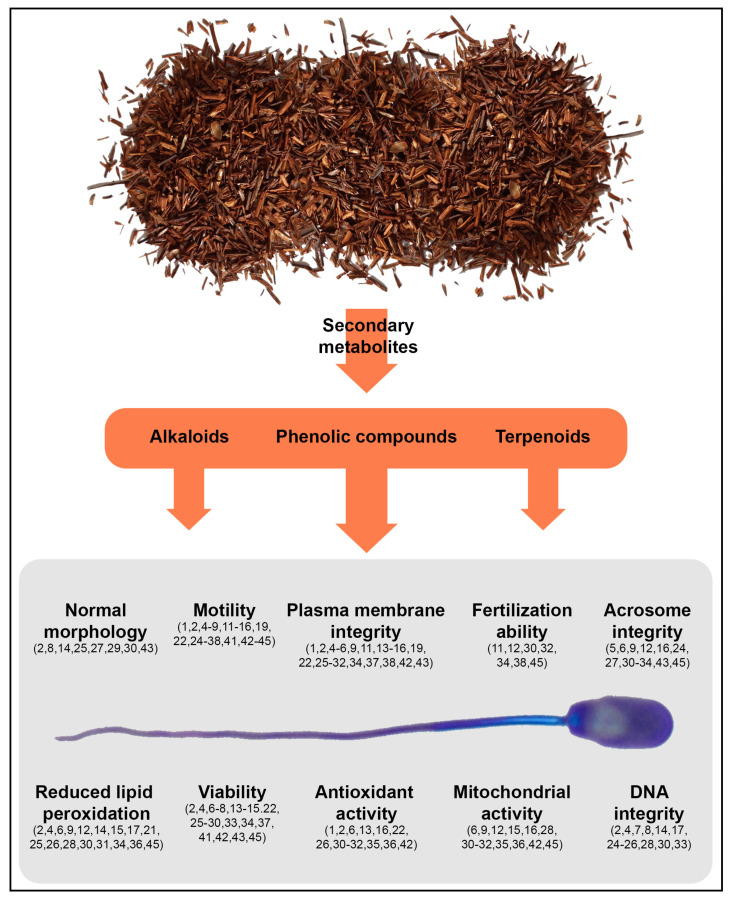
Positive effects of plant extracts on sperm parameters. The numbers within parentheses refer to the following plant species: *Achillea millefolium* (1), *Albizia harveyi* (2*), Aloe vera* (3), *Argania spinosa* (4), *Aspalathus linearis* (5), *Camellia sinensis* (6), *Capparis spinosa* (7), *Ceratonia siliqua* (8), *Cyclopia intermedia* (9), *Cymbopogon citratus* (10), *Diospyros kaki* (11), *Echinacea purpurea* (12), *Entada abyssinica* (13), *Eurycoma longifolia* (14), *Foeniculum vulgare* (15), *Gynostemma pentaphyllum* (16), *Ilex paraguariensis* (17), *Lippia origanoides* (18), *Lolium perenne* (19), *Melaleuca alternifolia* (20), *Melissa officinalis* (21), *Moringa oleifera* (22), *Myrrhinium atropurpureum* (23), *Nigella sativa* (24), *Opuntia ficus-indica* (25), *Origanum vulgare* (26), *Phoenix dactylifera* (27), *Portulaca oleracea* (28), *Psidium guajava* (29), *Punica granatum* (30), *Rhodiola rosea* (31*), Salvia miltiorrhiza* (32*), Salvia officinalis* (33), *Salvia rosmarinus* (34), *Sambucus nigra* (35), *Schisandra chinensis* (36), *Syzygium aromaticum* (37), *Syzygium cumini* (38), *Thymus capitatus* (39), *Thymus munbyanus* (40), *Tribulus terrestris* (41), *Ugni molinae* (42), *Urtica dioica* (43), *Viscum album* (44), *Zingiber officinale* (45).

Tens of plant species (extracts) have been used as preservatives for semen storage in several animal species, including humans. The extracts are usually added to the semen extender and used both for sperm refrigeration and cryopreservation. These compounds can be incorporated into the diluents during all the steps involved in the sperm cryopreservation process (i.e., pre-freezing cooling/equilibration, and post-thawing incubation). Overall, sperm parameters and fertilization ability can be improved by the action of these natural compounds; in addition, some plant species can exert protection against pathogenic bacteria and act as ROS scavengers. In this section, a detailed description of the effects of 45 plant species on sperm function in several animal species is presented (Figure 1 and Figure 2). Moreover, the plant material and extraction method employed, dosage, possible toxic effects, and antimicrobial properties (Table 1), as well as a general introduction to the different plant families [27,28,29,30,31,32,33,34,35] of the plant species described here, are also provided.

#### 3.1.1. Family Adoxaceae

This group of plants comprises small trees, shrubs, and perennial herbs. The woody species are commonly found in gardens and some have edible fruits. Within the five genera found, the smallest (*Adoxa*, *Sinadoxa*, and *Tetradoxa*) are exclusively herbaceous, while the largest (*Viburnum* and *Sambucus*) are both woody and herbaceous. There are around 225 species worldwide, predominantly in temperate regions of Asia, Europe, and North America. The genus *Viburnum* also grows in tropical mountains. *Sambucus* spp., known as elderberries, accounts for 10 species (small trees and shrubs) and several of them produce fruits that are edible (if cooked). European elderberry (*Sambucus nigra*) is commonly used in herbal medicine.

##### *Sambucus nigra* (elder)

Bull spermatozoa were stored and supplemented with this fruit extract for a period of 24 h at room temperature. As a result, elder extract showed positive effects on sperm motility and mitochondrial activity together with a reduction in ROS production. The optimal concentrations of the extract were 5 and 10 µg/mL. On the other hand, the highest extract concentrations exerted a deleterious effect on sperm progressive motility and ROS production [36].

#### 3.1.2. Family Apiaceae

Commonly named umbellifers, the species from this group are grouped into more than 400 genera and 3500 species. The members of this vast family are present worldwide, being more abundant in temperate regions. While many species of this group are poisonous (e.g., poison hemlock), others are extensively used vegetables (parsley, carrot, celery, and fennel), spices, and herbs (e.g., anise, dill, coriander, caraway, cumin).

##### *Foeniculum vulgare* (fennel)

This plant has been used as an additive for boar [37] and ram [38] semen diluents during sperm cryopreservation. In boar, ejaculated sperm were frozen by using three concentrations of fennel (2.5, 5, and 10 g/100 mL). The plant extract had positive effects on sperm motility and viability, especially at the highest concentration tested. Moreover, the two highest concentrations reduced the malondialdehyde (MDA) content of the sperm samples when compared to the control group. In ram, semen was cryopreserved by the use of three extract concentrations: 5, 10, and 15 mg/mL. Fennel improved several sperm parameters, such as viability, motility, kinetics, plasma membrane integrity, and mitochondrial activity, and reduced the MDA content. The most suitable extract concentration for the cryopreservation of ram semen was 10 mg/mL at a concentration of 200 × 10^6^ sperm/mL.

#### 3.1.3. Family Aquifoliaceae

This family is also known as the holly family. The holly genus *Ilex* comprises more than 400 species worldwide. The majority of species grow in mountainous areas of the tropics, mainly South-Central America and Asia. Generally, the family included shrubs or trees, most of which are evergreen. The leaves of the well-known *I. paraguariensis* are used in South America to prepare the well-known tea called yerba mate. The most representative species in Europe is the European holly (*I. aquifollium*), widely used for decoration.

##### *Ilex paraguariensis* (yerba mate)

This plant extract has been used as an additive in the cryopreservation process of boar epididymal sperm [39]. The tested concentrations ranged from 2.5 to 10 g/L. Within all the sperm parameters assessed after thawing, the mate tea reduced lipid peroxidation (MDA levels) and protected DNA against oxidative damage. The best results were obtained by using an extract concentration of 10 g/L in the extender.

#### 3.1.4. Family Arecaceae

This is also called the palm family, which contains approximately 2400 species. Despite being considered a tropical family, palms can be found worldwide, except in Antarctica. The northernmost palm is the European fan palm (*Chamaerops humilis*; Mediterranean areas in Europe and North Africa) and the southernmost is the nikau palm (*Rhopalostylis sapida*; New Zealand). Among the most recognized species are coconut, date, and oil palms.

##### *Phoenix dactylifera* (date palm)

Pollen from this palm has been used as an additive during chilling (buffalo and bull sperm) and cryopreservation (buffalo and stallion sperm). The extract was added to semen extenders with or without egg yolk. After 7 days of buffalo semen chilling and after semen thawing [40], the authors observed significant improvements in sperm motility, viability, and membrane integrity. The best results were obtained using a set of concentrations ranging from 10 to 50 mg/mL. However, some treatments showed deleterious effects on sperm function. Similarly, after stallion semen thawing [41], date palm extract exerted a protective effect on sperm motility and acrosome and membrane integrity at a range of concentrations from 20 to 30 mg/mL. On the other hand, some extract concentrations had negative effects on several sperm parameters. Moreover, the addition of this plant extract on bull semen [42] (both refrigerated at 5 °C and cryopreserved) exerted positive effects on sperm motility, viability, and plasma membrane integrity. Additionally, in the cryopreserved samples treated with this plant, there was a higher percentage of sperm with an intact acrosome and normal morphology than in the control group. The extract concentration employed was 0.4 mg/mL.

#### 3.1.5. Family Asphodelaceae

The majority of the members of this group belong to the Old World and it comprises around 900 species (Asia, Africa, and Europe). Only two genera are found in South America, while they are absent in polar regions and North America. More than half of the species from this family belong the genus *Aloe*, which has at least two species with medicinal and cosmetic uses. 

##### *Aloe vera* (aloe)

*A. vera* has been used as an alternative cryoprotectant to egg yolk for the chilling and cryopreservation of peccary semen [43]. The authors concluded that *A. vera* extract at a 20% concentration could be used as a substitute for egg yolk for the chilling and freezing of collared peccaries’ semen.

#### 3.1.6. Family Asteraceae

This is the largest dicot family, with approximately 25,000 species worldwide, and includes trees, shrubs, succulents, herbs, and vines, among others. This group is especially diverse in arid regions and Mediterranean-like climates and rarer in tropical rainforests. Among the most significant crop species are sunflower, lettuce, artichokes, and chicory. Many species are used as medicinal herbs (e.g., *Achillea* spp.) and insect deterrents (*Tanacetum coccineum*).

##### *Achillea millefolium* (yarrow)

This plant extract has been used as an additive in the rooster semen freezing extender both as a loaded nano-phytosome and as an extract alone [44]. The range of extract concentrations employed was from 1.5 to 4.5 mg/L. After thawing, this plant extract improved several sperm parameters (i.e., motility, kinetics, and membrane integrity), reduced the MDA content, and increased the total antioxidant capacity (TAC) and GPx activity. The best results were obtained at a concentration of 3 mg/L in nano-phytosome form directly included in the same extender.

##### *Echinacea purpurea* (echinacea)

The effects of this flowering plant have been tested in ram epididymal spermatozoa [45] by adding its extract to the freezing extender. The range of extract concentrations was from 5 to 20 mg/L. After thawing, several sperm parameters were enhanced by this natural additive, such as motility, kinetics, acrosome integrity, and mitochondrial activity. Moreover, the extract reduced the MDA content in the sperm samples and the cleavage rate was higher in oocytes fertilized with spermatozoa treated with this plant. On the other hand, the sperm samples treated with this plant (10 and 20 mg/L) showed higher ratios of sperm chromatin dispersion. All the extract concentrations used exerted positive results in at least one of the assessed sperm parameters.

#### 3.1.7. Family Cactaceae

This is a very popular family of plants that comprises more than 2000 species grouped into around 80 genera that almost strictly grow in the Americas. Within this group can be found succulent small trees, shrubs, and herbs typical of xeric habitats, with leaves normally reduced to spines. They are highly appreciated as ornamental plants and popular among hobbyists.

##### *Opuntia ficus-indica* (prickly pear)

Essential oil of this plant has been used as a supplement in tris egg yolk- and skim milk-based extenders during liquid storage (5 °C) of ram semen for 72 h [46]. The extract showed positive effects on sperm motility, viability, and morphology and reduced lipid peroxidation and DNA fragmentation during semen storage. For both extenders, the optimal concentrations were 1% and 2%. The use of acetone extract from cladodes of this plant, from the same species and under the same experimental conditions [47], improved sperm parameters (motility, viability, membrane integrity, and morphology) and reduced lipid peroxidation and DNA fragmentation during semen storage. However, the highest concentrations used (e.g., 4% and 8%) exerted deleterious effects on some sperm parameters when compared with the control group. The most suitable extract concentration was 1%.

#### 3.1.8. Family Capparaceae

These are trees, shrubs, and herbs widely distributed in warm temperate and tropical regions, usually with seasonal drought. This plant family is grouped into 17 genera with around 470 species. Eleven genera are endemic to certain regions: seven to Africa and four to Central-South America. While some species are grown as ornamentals, the pickled flower buds and fruits of *Capparis spinosa* are usually eaten (capers).

##### *Capparis spinosa* (caper bush)

The extract of this plant has been used as additive in human semen [48] incubated at 37 °C for 24 h. Three concentrations were used (15, 30, and 45 ppm). The sperm samples treated with this plant extract showed an enhancement in sperm parameters such as motility, velocity, and viability as well as a reduction in DNA fragmentation. These positive results were obtained with the highest extract concentrations (30 and 45 ppm).

#### 3.1.9. Family Crassulaceae

These are commonly named succulent stonecrops. These plants are typically evergreen herbs or shrubs, with a few being deciduous, annual, and aquatics. The family comprises approximately 1400 species, half of them belonging to the *Sedum* and *Crassula* genera. They are distributed worldwide, mostly inhabiting arid zones, but absent in polar regions and almost absent in tropical forests. 

##### *Rhodiola rosea* (golden root)

This plant extract has been used for the cryopreservation of boar semen [49]. The range of extract concentrations added to the semen extender was from 2 to 10 mg/L. After semen thawing, there was an enhancement in several sperm parameters, such as motility, acrosome and membrane integrity, and mitochondrial activity. An increase in GSH as well as a decrease in MDA levels were also observed in the sperm samples treated with this plant extract. The most suitable extract concentration ranged from 4 to 8 mg/L.

#### 3.1.10. Family Cucurbitaceae

This is commonly known as the squash family and is renowned for its swollen fruits (melons, zucchini, pumpkins, etc.). With more than 900 species and 100 genera, this plant group is widely known for its use as human food. Most are perennial herbaceous vines (prostrate or climbing) with spirally coiled tendrils. They are predominantly found in all tropical and subtropical regions worldwide and commonly grown in temperate regions around the world.

##### *Gynostemma pentaphyllum* (jiaogulan)

Boar spermatozoa were frozen using an extender supplemented with this plant [50] in a range of concentrations from 0.25 to 2 mg/mL. After semen thawing, there was an enhancement in several sperm parameters, such as motility, acrosome and membrane integrity, and mitochondrial activity. Related to biochemical assays, this plant extract also improved SOD levels. The most suitable extract concentration was 0.5 mg/mL. However, the highest extract concentrations exerted a significant decrease in several sperm parameters in comparison with the control group.

#### 3.1.11. Family Ebenaceae

The ebony family comprises more than 600 species, which are the source of several products with economic importance, such as timber (ebony) and fruits (e.g., persimmon). The family includes trees and shrubs, most of which are evergreen, and some are deciduous. The members of this group are distributed in pantropical regions, with a few species in temperate regions. The fruit is acutely astringent in all species until it is ripe.

##### *Diospyros kaki* (kaki)

The pulp of this plant exerted beneficial effects on bull sperm parameters during semen chilling and after freezing and thawing [51]. The extract concentrations employed ranged from 1% to 10%. There was an improvement in sperm motility during 11 days of semen storage at 5 °C. After semen thawing, this plant showed positive effects on sperm motility and membrane integrity. The best results on sperm parameters were obtained through the use of concentrations ranging from 1% to 6%. Moreover, a higher conception rate was observed in cows inseminated with semen samples treated with this pulp fruit at concentrations of 2%, 4%, and 6%.

#### 3.1.12. Family Fabaceae

Known as the legume family, this is a group of plants commonly found in vegetable gardens worldwide. Among the most commercially important legumes are lentils, peas, chickpeas, soy, and forage crops such as alfalfa and clover. With approximately 20,000 species and more than 700 genera, it is the third-largest plant family and its members can be found worldwide (except in polar regions), being especially diverse in the tropics. Herbs to trees commonly with nitrogen-fixing bacteria nodules on the roots. Some species (clover, alfalfa, etc.) are often used in crop rotation because they increase soil nitrogen.

##### *Albizia harveyi* (silk tree)

The effects of *A. harveyi* have been tested in bull sperm [52] by adding the extract to the semen extender prior the cryopreservation process. After semen thawing, a significant reduction in the MDA content and an increase in TAC were observed in the samples treated with this plant. Furthermore, the extract exerted protective effects on sperm membrane and DNA integrity, motility, and viability and reduced the percentage of sperm abnormalities. A reduction in the percentage of apoptotic sperm and ultra-structure damage was also observed. The suitable range of concentrations to be used on bull sperm is within 0.5–1.5 µg/mL.

##### *Aspalathus linearis* (rooibos)

This plant has been used in boar semen samples stored for 96 h at 17 °C [53]. Two types of rooibos (fermented and unfermented) were used. The range of extract concentrations employed was from 20 to 500 µg/mL. Even though the unfermented form had higher antioxidant capacity and total phenolic content than the fermented type, the latter showed the best effects on sperm function. During semen storage, this plant extract preserved sperm velocity, motility, and plasma membrane and acrosome integrity. The highest concentrations used significantly decreased total sperm motility without compromising acrosome and membrane integrity. The most suitable concentrations for boar semen were 20 and 60 µg/mL.

##### *Ceratonia siliqua* (carob)

This plant extract has been employed in human spermatozoa [54] by adding it to the freezing and thawing medium. The range of concentrations used was from 5 to 30 µg/mL. The addition of this plant to the semen extender significantly increased sperm progressive motility, normal morphology, and viability together with a reduction in DNA damage. However, some of the concentrations employed had deleterious effects on sperm parameters. The best results were obtained with a concentration of 10 and 20 µg/mL. 

##### *Cyclopia intermedia* (honeybush)

Honeybush has been tested as an alternative additive for boar semen preservation for 5 days at 17 °C [55]. In general, this plant extract was able to improve the preservative properties of a long-term semen extender. The positive results on sperm parameters were observed from 48 to 120 h of semen storage. An extract concentration of 12.5 µg/mL in semen samples simultaneously preserved sperm motility, kinetics, acrosome integrity, and mitochondrial activity at 120 h of semen storage. None of the concentrations used (12.5, 25, 50, and 200 µg/mL) were detrimental for sperm function.

##### *Entada abyssinica* (Splinter bean)

This plant has been used as an extender additive for ram semen cryopreservation [56]. A wide set of concentrations was used (125, 250, 375, and 500 µg/mL). After semen thawing, this plant extract exerted positive effects on several sperm parameters (motility, viability, and membrane integrity) as well as a reduction in the concentration of H_2_O_2_ of treated samples compared with the untreated samples. The most suitable concentration was 375 µg/mL. On the other hand, the extract concentration of 500 µg/mL had a negative effect on sperm viability.

#### 3.1.13. Family Lamiaceae

These are annual or perennial herbs, vines, shrubs, and trees distributed worldwide, being especially abundant in dry and hot regions such as Mediterranean areas. Additionally known as the mint family, the species from this group usually have square stems, opposite leaves, and aromatic essential oils. The evaporation of essential oils reduces water loss in xeric areas. Widely used as culinary and medicinal herbs, this family comprises around 7000 species grouped into more than 250 genera. Among the most popular genera are *Thymus*, *Salvia*, *Mentha*, *Lavandula*, and *Origanum*.

##### *Melissa officinalis* (lemon balm)

When added to the freezing media, this plant extract enhanced the linearity (LIN) and straightness (STR) of sperm trajectory just after boar semen thawing [39]. During sperm incubation for 120 min at 37 °C, there were no significant effects on sperm kinetics. Otherwise, lemon balm reduced the MDA content in the semen samples after thawing. The most suitable concentration for boar semen is within 2.5–5 g/L.

##### *Origanum vulgare* (oregano)

This plant extract has been employed as an additive for semen preservation in bull [57], human [58], and rabbit [59]. Different results were obtained within the species tested. In bull, this plant has been used in the semen cryopreservation process. The extract was added to the sperm freezing medium. Several extract concentrations were employed ranging from 2 to 20 mL/dL. After semen thawing, several sperm parameters (motility, viability, and membrane integrity) were enhanced by the action of this plant. There was also a reduction in the MDA content and increased activity of SOD and CAT in the sperm samples. However, the highest concentrations (16 and 20 mL/dL) exerted negative effects on some sperm parameters. The most suitable concentrations for cryopreserved bull semen were 2 and 4 mL/dL. During incubation (5 and 10 min) of human semen samples, this plant extract (1.5 µL of oil extract in around 500 µL of diluted sperm) exerts positive effects on several sperm velocity parameters such as curvilinear velocity (VCL), straight-line velocity (VSL), average path velocity (VAP), and amplitude of lateral head displacement (ALH). Furthermore, after 10 min of semen incubation, this extract showed a protective effect on sperm DNA integrity. In rabbit, this plant extract was tested during fresh semen incubation at room temperature. While the lowest concentrations used (37.5 and 75 µg/mL) did not have positive or negative results on any of the sperm parameters evaluated, the highest concentrations (150 and 300 µg/mL) were detrimental for sperm function.

##### *Salvia miltiorrhiza* (red sage)

This plant has been used as an additive to the semen extender prior to boar sperm freezing at five extract concentrations ranging from 0.2 to 1 mg/mL [60]. After semen thawing, there was an enhancement in several sperm parameters (motility, membrane and acrosome integrity, and mitochondrial activity), enzymatic activities (SOD; lactate dehydrogenase, LDH; glutamic-oxaloacetic transaminase, GOT; and CAT), and fertility outcomes (pregnancy rate and litter size). The optimal concentration for boar semen cryopreservation was 0.4 mg/mL.

##### *Salvia officinalis* (sage)

The addition of this plant extract to the boar semen extender improved several sperm parameters after freezing and thawing [61]. The extract concentrations employed were 2.5, 5, and 10 g/L. During the pre-freezing process, this plant had a positive effect by increasing the progressive sperm motility. Moreover, after semen thawing, the sage extract enhanced sperm viability and protected acrosome and DNA integrity. The most suitable concentration of sage for boar sperm cryopreservation was 5 g/L.

##### *Salvia rosmarinus* (rosmarinus)

Rosemary has been used as an additive for the preservation of boar, goat, ram, and rooster sperm. In boar [62], rosemary has been used as an additive for semen cryopreservation at a concentration of 1 mg/mL. Several sperm parameters were evaluated at 0, 1, 2, and 3 h after thawing of semen incubated at 37 °C. Rosemary extract exerted positive effects on sperm viability, motility, and plasma membrane and acrosome integrity. In addition, the sperm samples treated with this plant showed better performance on in vitro fertilization trials. In the same species [63,64], this plant has been used in fresh semen incubated at 16 °C for 3 h. Ten extract concentrations were added to the semen extender, ranging from 0.2 to 2 mg/mL. While some concentrations did not significantly alter sperm parameters (up to 0.6 mg/mL), the highest concentrations (starting from 0.8 mg/mL) had a negative impact. On the other hand, even though a concentration of 0.4 mg/L did not show an enhancement on sperm parameters, it exerted an antimicrobial effect similar to that of ampicillin against *Escherichia coli*. In goat [65], this plant extract has been used for semen cryopreservation at 2%, 4%, and 6%. Positive effects were found on sperm viability, motility, and plasma membrane integrity. Moreover, an extract concentration of 2% reduced the MDA content in the sperm samples when compared with the control group. In general, the 4% concentration showed the best effects on sperm function. However, the highest extract concentration (6%) exerted negative effects on some sperm parameters. Similar results were obtained in ram [66], where the semen was cryopreserved by using four concentrations of rosemary extract (2%, 4%, 6%, and 8%). Thus, this plant extract enhanced several sperm parameters, such as viability, motility, and plasma membrane integrity, and reduced the MDA content. The best results were obtained by the use of 4% and 6% of plant extract, whereas the highest (8%) concentration had deleterious effects on some sperm parameters when compared with the control group. Finally, rosemary extract was used as an additive for the preservation of rooster semen [67] at 4 °C for 48 h. The sperm motility and kinetics were improved during semen storage. The best results were obtained by using a concentration of 8.7 and 87 µg/mL (especially at 6 h of semen storage), while a concentration of 870 µg/mL was detrimental for sperm function.

##### *Thymus capitatus* (Spanish oregano)

The extract of this plant has been used in boar semen [63] incubated at 16 °C for 3 h. Ten extract concentrations were added to the semen extender, ranging from 0.2 to 2 mg/mL. As a result, this plant impaired sperm function starting from the lowest concentration and showed spermicidal effects from 0.4 mg/mL.

##### *Thymus munbyanus* (thyme)

The extract of this thyme species has been used in human semen [68] incubated for 30 min. This extract impaired human sperm motility and its capacity to undergo hyperphosphorylation and acrosome reaction. The range of concentrations tested was 100–1000 µg/mL.

#### 3.1.14. Family Lythraceae

This is a group that is widely distributed but predominately found in tropical areas and less so in temperate regions. Plants are annual or perennial, mostly herbs, sometimes shrubs, and rarely trees, with approximately 30 genera and 600 species. They include several dye plants, such as *Lawsonia inermis* (henna), and fruit trees (e.g., *Punica granatum*; pomegranate). Members of *Cuphea* have been identified as a good source of medium-chain triglycerides, while other genera provide good-quality timber (e.g., *Physocalymma scaberrimum*).

##### *Punica granatum* (pomegranate)

The fruit juice of this plant has been employed for the cryopreservation of buffalo [69], bull [70], and ram [71] semen. In buffalo, the semen extender was supplemented with a range of concentrations from 2.5% to 10%. Even though the best results were obtained with a 10% concentration of juice, all the tested extract concentrations enhanced several sperm parameters, such as motility, velocity, normal morphology and integrity of plasmalemma, acrosome, and DNA. Furthermore, under in vivo fertility trials, the use of semen samples supplemented with a 10% concentration of this plant extract led to higher pregnancy rates when compared with the control group. In bull, pomegranate extract has been added to the semen extender for sperm cryopreservation. A range of concentrations from 10% to 50% was used. There was an improvement in sperm motility and viability after semen thawing with the use of the lowest extract concentrations (10% and 20%). By contrast, the highest concentrations showed deleterious effects on sperm parameters. After ram semen thawing, several sperm parameters (motility, membrane integrity, viability, and mitochondrial activity) were enhanced by the use of this plant at a concentration of 5 mg/L. At the same concentration, there was also a reduction in MDA levels and an increased TAC in the semen samples. However, several sperm parameters were negatively affected at the highest concentration used (7.5 mg/L).

#### 3.1.15. Family Moringaceae

This is a plant family mainly composed of trees, shrubs, and subshrubs with soft and usually brittle wood. This monogeneric (*Moringa*) group contains 13 species primarily distributed in the Horn of Africa but also on the southern coast of the Arabian Peninsula, India, Madagascar, Angola, and Namibia. Even though all the species have local economic uses, *M. oleifera* is the species with major interest due to its widespread use in folk medicine and as a leaf vegetable crop.

##### *Moringa oleifera* (moringa)

This plant extract has been used for the cryopreservation of banana shrimp spermatophores and ram spermatozoa. In banana shrimp [72], moringa extract had no detrimental or positive effects on sperm function after spermatophore thawing. However, the addition of this extract to the sperm extender promoted a reduction in bacterial abundance in cryo-stored spermatophores. The extract concentration used was 0.1 mg/mL. This plant extract (0.1 mg/mL) has been also employed during cool storage (2–4 °C for 28 days) of banana shrimp spermatophores [73]. This plant exerts positive effects on sperm survival together with the complete elimination of several bacterial contaminants. In ram [74], the addition of this plant to the freezing extender exerted positive effects on several sperm parameters after semen thawing. Thus, an extract concentration within 0.5–5 mg/mL had a protective effect on sperm motility and membrane integrity together with increased antioxidant activity of the semen samples. The best results were obtained with the lowest extract concentration (0.5 mg/mL).

#### 3.1.16. Family Myrtaceae 

Additionally known as the myrtle family, this family comprises more than 5500 species grouped into approximately 140 genera. Typically, they grow in tropical and warm-temperate regions and are particularly diverse in Australia and tropical America. Woody shrubs and trees, mostly evergreen, with exotic-looking flowers and essential oils. Among the most popular genera are *Melaleuca* (tea tree), *Psidium* (guava), *Syzygium* (clove), *Eucalyptus*, and *Myrtus*.

##### *Melaleuca alternifolia* (tea tree)

The essential oil of this plant has been tested on boar spermatozoa [64,75] incubated at 16 °C for 3 h at a range of concentrations from 0.2 to 2 mg/mL. While some concentrations did not significantly affect sperm parameters, others had a negative impact. On the other hand, even though a concentration of 0.4 mg/L did not show an enhancement of sperm parameters, it exerted a similar antimicrobial effect to that of ampicillin in boar semen stored at 16 °C for 120 h.

##### *Myrrhinium atropurpureum* (palo de fierro)

The extract of this plant was added to the extender for boar semen storage [76] at 17 °C. Among the four concentrations used, two of them (0.1% and 1%) showed cytotoxic effects immediately after their addition to the culture media. A concentration of 0.01% had deleterious effects on sperm parameters after 2 h of semen incubation. The remaining concentration employed (0.001%) had neither positive nor negative results on sperm function. None of the concentrations tested had inhibitory effects on bacterial growth (*Escherichia coli* and *Pseudomonas aeruginosa*).

##### *Psidium guajava* (common guava)

Leaf tannins from common guava have been added to the semen extender for the preservation of Etawa goat sperm [77] for 15 days at 4–5 °C. The range of concentrations employed was from 3% to 24%. The supplementation of the semen extender with guava extract improved sperm motility, viability, and plasma membrane integrity and reduced the percentage of sperm abnormalities. The most suitable extract concentration was 3%. By contrast, an extract concentration from 6% to 24% was deleterious for some sperm parameters.

##### *Syzygium aromaticum* (clove)

Clove bud has been employed as an additive to different types of extenders for ram sperm cryopreservation [78]. The tested extract concentrations ranged from 35 to 115 µg/mL. Sperm motility and kinetics, viability, and membrane integrity were better preserved by this plant extract during semen cooling and after thawing in comparison with the control group. The suitable range of extract concentrations was from 35 to 75 µg/mL. On the other hand, a concentration of 115 µg/mL had deleterious effects on sperm function.

##### *Syzygium cumini* (Jambul)

The fruit pulp extract of this plant has been used for the cryopreservation of bull semen [79] at two concentrations (7 and 14 ppm). Both concentrations of this plant extract enhanced sperm motility, membrane integrity, and fertilization ability.

##### *Ugni molinae* (Chilean guava)

This plant extract has been used as an additive for boar semen storage at 17 °C for 7 days [80]. The extract concentration employed was 0.315 µg/mL. During semen storage, there was an improvement in the sperm motility, viability, and mitochondrial activity together with a reduction in the ROS production in the samples treated with this plant.

#### 3.1.17. Family Poaceae

The grass family is one of the most prominent groups that has great economic importance to humans (food crops, shelter, fodder, industry, decontamination, etc.). There are more than 10000 species grouped into around 800 genera. Corn, rice, wheat, and bamboo are part of this family, which is distributed worldwide and represents approximately 20% of the Earth’s vegetative cover. Grasses are usually annual or perennial herbs. Even though they are not truly woody, like dicot trees or shrubs, some canes have similar strength to steel.

##### *Cymbopogon citratus* (lemon grass)

The essential oil of this plant has been tested on boar sperm during 2 days of semen storage at 17 °C [76]. All the concentrations used (0.001-1%) were detrimental for sperm parameters. The authors concluded that the essential oil from this plant is unsuitable for boar sperm preservation due to its cytotoxicity. In addition, the concentrations tested had no inhibitory effect on *Escherichia coli* and *Pseudomonas aeruginosa* growth.

##### *Lolium perenne* (rye grass)

Isolated proteins from this frost-resistant plant (0.1, 1, and 10 µg/mL) have been used as an additive to cryopreserve Nili-Ravi buffalo sperm [81]. After semen thawing, samples treated with this plant (0.1 and 1 µg/mL) showed higher sperm progressive motility and plasma membrane integrity than the control group. None of the concentrations used were detrimental for sperm function.

#### 3.1.18. Family Portulacaceae

This group of plants comprises succulent annual or perennial herbs to shrubs with mucilaginous tissues. This family is widely distributed in warm and temperate regions and is especially diverse in the Americas and Southern Africa, being adapted to dry areas of high light intensity. There are around 19 genera and 500 species, among which the genus *Portulaca* comprises around 200 species. The members of this family are well known for their medicinal properties and also commonly found as ornamentals in gardens.

##### *Portulaca oleracea* (pursley)

This plant extract has been employed for goat semen cryopreservation [82]. Different extraction methods and concentrations (25, 50, and 100 µg/mL) were used. Pursley extract had positive effects on several sperm parameters (motility, viability, mitochondrial activity, plasma membrane and DNA integrity) and reduced the MDA content in the semen samples when compared to the control group. The most suitable concentration was 50 µg/mL and the best results were obtained with the methanolic extract. However, some concentrations (from different extraction methods) were detrimental for sperm parameters.

#### 3.1.19. Family Ranunculaceae

These are known as the buttercup family and are predominantly herbaceous annual and perennial plants. The family includes also some woody vines and prefers moist habitats. They commonly have rhizomes or tubers. Several alkaloids, glycosides, and saponins are frequently found in the sap. There are more than 1800 species and around 50 genera, mainly found in temperate and cold areas of the northern hemisphere. The group is widely distributed as ornamental plants in gardens. Some of the most popular genera are *Aconitum*, *Aquilegia*, *Anemone*, *Ranunculus*, and *Nigella*.

##### *Nigella sativa* (black caraway)

Seminal doses from goat [83] and ram [84] were treated with this plant. In goat, this extract was tested both in fresh and cryopreserved semen at a concentration of 0.5%. In fresh semen, *N. sativa* had negative effects on sperm motility after 1.5 and 2 h of sperm collection, while progressive motility and sperm abnormalities were not significantly different from the control group. In the cryopreserved samples, there were neither positive nor negative effects on sperm function. In ram, this plant was added to the semen extender before sperm cryopreservation. The extract concentrations employed ranged from 10 to 1000 µg/mL. Immediately after semen thawing and at 2 h of post-thaw incubation, there were enhancements in several sperm parameters, such as motility, kinetics, and acrosome and DNA integrity. The most suitable concentration for the cryopreservation of ram semen was 100 µg/mL. However, the highest extract concentration had deleterious effects on some sperm parameters.

#### 3.1.20. Family Santalaceae

Commonly known as sandalwoods, this family includes more than 1000 species grouped into around 40 genera distributed worldwide. Half of the genera are found in dry or temperate areas, while the other half are found in tropical rainforests. The group includes perennial herbs, epiphytic climbers, shrubs, and small trees. Most species are hemiparasitic towards other plants (e.g., Mistletoe; aerial parasitism).

##### *Viscum album* (Mistletoe)

The extract of this parasitic plant has been added to rabbit semen during incubation at 37 °C for 3 h [85]. The range of concentrations used was from 2 to 10 mg/mL. Only at the initial time of sperm incubation (0 h) was an increase in sperm motility and velocity observed. Moreover, during the following incubation times, the lowest concentrations did not show any positive effects, but detrimental effects were observed at the highest concentrations used in several sperm parameters.

#### 3.1.21. Family Sapotaceae

This family includes around 1000 species of trees or shrubs grouped into approximately 50 genera that inhabit pantropical regions, especially in lowlands and montane rainforests. The members of this family are mostly evergreen plants that usually produce edible fruits and milky sap. Some of the most popular species are *Vitellaria paradoxa* (shea tree) and *Argania spinosa* (Argan).

##### *Argania spinosa* (argan)

*A. spinosa* has been employed as an additive in ram semen [86] during liquid storage at 5 °C and 15 °C during 48 h. The range of concentrations employed was from 1% to 10% (*v/v*). Argan oil improved several sperm parameters, such as viability, progressive motility, and membrane integrity, and reduced lipid peroxidation levels (MDA) and DNA fragmentation index. Nevertheless, some concentrations used were detrimental for membrane integrity at different storage times. The best results on sperm parameters were obtained with concentrations from 1% to 5%.

#### 3.1.22. Family Schisandraceae

This is a small group of plants with approximately 50 species belonging to two genera. They are climbers and aromatic plants. All the species are found in Southeastern Asia, except for one species from the genus *Schisandra* that inhabits the Southeastern United States.

##### *Schisandra chinensis* (omija)

This plant has been tested on bull semen [87] stored for 24 h at room temperature (22–25 °C). Within the different concentrations tested (1–75 µg/mL), those within the range of 5–50 µg/mL showed antioxidant properties and exerted the best effects on sperm parameters. Thus, sperm motility and mitochondrial activity were enhanced by this plant extract. Moreover, lipid peroxidation, ROS production levels, and protein oxidation were significantly lower in the samples treated with this plant extract in comparison with the control group. None of the concentrations employed exerted negative effects on sperm function. Moreover, the omija extract exerted antibacterial activity against several species, being more effective against Gram-positive bacteria.

#### 3.1.23. Family Simaroubaceae

This family includes 32 genera and around 200 species mainly distributed in pantropical and a few species in subtropical and temperate regions. The group includes trees and shrubs, with several species widely employed in folk medicine to treat malaria, viral diseases, diabetes, and gastritis, among others. The most popular species from this family is the tree of heaven (*Ailanthus altissima*), which is considered a noxious and invasive tree that has widely colonized Europe and North America.

##### *Eurycoma longifolia* (tongkat ali)

This plant has been used in chilled (5 °C for 6 days) and cryopreserved bull sperm [88]. The extract concentration in the semen extender ranged from 0.25 to 7.5 mg/mL. For both processes, the extract exerted positive effects on sperm motility, viability, and membrane integrity. Additionally, in the chilled samples, there was a reduction in the percentage of sperm with abnormal morphology. After semen thawing, there was also a reduction in the MDA levels and DNA damage in the samples treated with this plant. In the chilled samples, the best results were obtained with extract concentrations of 0.25 and 1 mg/mL, while in the frozen samples, a concentration of 5 mg/mL was the most suitable. However, the highest concentrations employed exerted deleterious effects on some sperm parameters.

#### 3.1.24. Family Theaceae

This is widely known as the source of tea leaves (*Camellia sinensis*). It is a family of trees and shrubs grouped into approximately 250 species and around 28 genera, mainly found in tropical and temperate regions of East- and Southeastern Asia and Eastern North America through the Caribbean to South America. Several species are grown as ornamentals, mainly for their handsome, fragrant, and showy flowers, such as *Stewartia* and *Polyspora*. Saponins, alkaloids, tannins, and calcium oxalate crystals are usually found in the main species.

##### *Camellia sinensis* (tea plant)

This plant has been widely used for sperm preservation in a large number of animal species, such as boar [89,90,91], dog [92], mouse [93], and ram [94]. Moreover, both fermented (black) and unfermented (green and white) types have been used with satisfactory results. In boar, green tea extract has been employed for the cryopreservation of semen. In this study [89], Gale et al. found reduced sperm MDA levels in the presence of tea at a concentration of 5%, although other concentrations showed detrimental effects on sperm parameters. The range of concentrations used was from 2.5% to 10%. By contrast, other authors [90,91] using a range of concentrations from 0.01% to 0.2% found positive effects on several sperm parameters (viability, motility, and acrosome integrity) as well as in the in vitro maturation of oocytes. However, the highest extract concentrations employed had deleterious effects on some sperm parameters. In dog [92], green tea has been tested for long-term semen storage (four weeks at 5 °C). The supplementation of semen with extract concentrations ranging from 0.5 to 1 mg/mL increased sperm motility and viability during the storage period. White and green tea have been tested on mouse epididymal spermatozoa [93] stored at room temperature for 72 h. Both extracts enhanced several sperm parameters during the incubation period. Two extract concentrations were used (0.5 and 1 mg/mL). The samples treated with this plant showed higher antioxidant potential and sperm viability together with a reduction in the MDA levels when compared with the control group. The white type showed better results in all the parameters evaluated, with the concentration of 1 mg/mL being the most suitable. Green tea has been also employed for the cryopreservation of ram semen [94] by adding to the semen extender three extract concentrations (5, 10, and 15 mg/L). This plant extract exerted positive effects on sperm viability, motility, plasma membrane integrity, and mitochondrial activity. Moreover, the samples treated with this plant showed higher antioxidant capacity and lower levels of MDA than the control group. However, the highest concentrations used promoted an increase in the MDA levels (15 mg/L) and a decrease in the activity of GPx (10 mg/L) in the semen samples. The best results were obtained with an extract concentration of 10 mg/L.

#### 3.1.25. Family Urticaceae

This plant family is distributed worldwide in a wide range of habitats. The group includes herbs, shrubs, and trees, usually with small greenish flowers, with some species bearing stinging hairs. They are grouped into around 43 genera and 1700 species. Members of this family are commonly used as a source of fiber for clothing (*Boehmeria* spp.), gardening (*Pilea* spp.), and food (*Urtica* spp.).

##### *Urtica dioica* (common nettle)

This plant has been tested in cryopreserved and refrigerated (5 °C) bull semen [42]. The extract concentration employed was 200 µg/mL. Positive effects were observed in both cryopreserved and refrigerated sperm samples. More specifically, this plant extract enhanced sperm motility, viability, and acrosome and membrane integrity. There was also a reduction in the percentage of abnormal sperm in the cryopreserved samples.

#### 3.1.26. Family Verbenaceae

This is a group of plants mainly found in tropical and subtropical regions, with a few representatives in temperate regions (herbs). The family usually comprises aromatic herbs, shrubs, or, rarely, trees or vines, often armed with prickles or thorns. There are approximately 1000 species grouped into more than 30 genera, usually with essential oils and calcium oxalate crystals in their tissues. The family has ornamental (*Congea* spp.), timber (*Citharexylum* spp.), edible (*Priva* spp.), and medicinal species (*Verbena* spp.).

##### *Lippia origanoides* (salva-de-Marajó)

The addition of this plant to the freezing extender in buffalo [95] did not enhance any sperm parameter after semen thawing. The extract concentrations employed were 2.5, 5, and 10 µg/mL.

#### 3.1.27. Family Zingiberaceae

There are perennials, aromatic herbs with tuberous rhizomes, and secretory cells with ethereal oils. With around 1000 species and 50 genera, this plant group grows in pantropical areas and is especially diverse in Southeastern Asia, typically in shady, lowland forests with rich soil. Among the most popular species are ginger, cardamom, and turmeric. Within this plant family, there are numerous ornamental species (torch ginger, golden brush, dancing girl, etc.).

##### *Zingiber officinale* (ginger)

The rhizomes of this plant have been used as additives for the cryopreservation of ram epididymal sperm [45] and shrimp spermatophores. Ram sperm were cryopreserved by using a Tris egg yolk diluent supplemented with three concentrations of ginger extract (5, 10, and 20 mg/L). After sperm thawing, several parameters (viability, motility, velocity, acrosome integrity, and mitochondrial activity) were enhanced by this plant extract when compared with the control group. Moreover, the MDA levels were lower in the samples treated with this plant. The best results were obtained by the addition of 5 and 10 mg/L of this plant extract to the semen extender. Moreover, the cleavage rate was markedly higher in matured oocytes fertilized with frozen semen treated with 20 mg/L. On the other hand, the sperm samples treated with this plant showed a higher ratio of sperm chromatin dispersion. In shrimp [72], even though the ginger extract (0.1 mg/mL) did not exert either positive or negative effects on sperm survival, supplementation with this plant led to a reduction in bacterial abundance in cryo-stored spermatophores.

#### 3.1.28. Family Zygophyllaceae

There are herbs, shrubs, and, rarely, trees that usually grow in xeric and saline areas. This plant group comprises approximately 250 species grouped into around 25 genera that are distributed mainly in tropical to subtropical zones, with some representatives in temperate areas, especially in dry Mediterranean regions. Within this family, there are timber and medicinal species (*Guaiacum* spp.) and several grown as ornamental plants (*Tribulus* spp. and *Zygophyllum* spp.).

##### *Tribulus terrestris* (calthrop)

In humans, this plant extract has been added to the semen extender before sperm cryopreservation and after thawing [96]. An improvement in sperm motility and viability was detected when it was added after semen thawing. The concentrations used were 20, 40, and 50 µg/mL, and the best results were obtained with the two highest concentrations. None of the concentrations used had deleterious effects on sperm parameters.

**Table 1:** Extraction methods, part of the plant used, toxicity, and antimicrobial activity of the plant species (*N* = 45) tested in the different animal species.

Plant Species (Family)	Animal Species [References]	Extraction Method	Plant Part	Toxicity	Antimicrobial Activity
*Achillea millefolium* (Asteraceae)	Chicken [44]	Water	Aerial parts	No	NE
*Albizia harveyi* (Fabaceae)	Cattle [52]	Methanol	Leaves	No	NE
*Aloe vera* (Asphodelaceae)	Peccary [43]	Pulp	Leaves	No	NE
*Argania spinosa* (Sapotaceae)	Sheep [86]	Oil	Seeds	DD	NE
*Aspalathus linearis* (Fabaceae)	Pig [53]	Water	Leaves, stems	DD	NE
*Camellia sinensis* (Theaceae)	Dog, mouse, pig, sheep [89,90,91,92,93,94]	Water	Leaves, powder	DD	NE
*Capparis spinosa* (Capparaceae)	Human [48]	Ethanol	Leaves	No	NE
*Ceratonia siliqua* (Fabaceae)	Human [54]	Water	Not specified	DD	NE
*Cyclopia intermedia* (Fabaceae)	Pig [55]	Water	Leaves, stems	No	NE
*Cymbopogon citratus* (Poaceae)	Pig [76]	Oil	Leaves	Toxic	No
*Diospyros kaki* (Ebenaceae)	Cattle [51]	Pulp	Pulp (fruit)	No	NE
*Echinacea purpurea* (Asteraceae)	Sheep [45]	Water	Leaves	No	NE
*Entada abyssinica* (Fabaceae)	Sheep [56]	Methanol	Bark	DD	NE
*Eurycoma longifolia* (Simaroubaceae)	Cattle [88]	Water	Not specified	DD	NE
*Foeniculum vulgare* (Apiaceae)	Pig, sheep [37,38]	Water	Leaves, seeds	No	NE
*Gynostemma pentaphyllum* (Cucurbitaceae)	Pig [50]	Several steps	Not specified	DD	NE
*Ilex paraguariensis* (Aquifoliaceae)	Pig [39]	Water	Leaves	No	NE
*Lippia origanoides* (Verbenaceae)	Buffalo [95]	Oil	Not specified	No	NE
*Lolium perenne* (Poaceae)	Buffalo [81]	Several steps	Leaves	No	NE
*Melaleuca alternifolia* (Myrtaceae)	Pig [64,75]	Oil	Not specified	DD	Yes
*Melissa officinalis* (Lamiaceae)	Pig [39]	Water	Leaves	No	NE
*Moringa oleifera* (Moringaceae)	Sheep, shrimp [72,73,74]	Ethanol, methanol	Leaves, seeds	No	Yes
*Myrrhinium atropurpureum* (Myrtaceae)	Pig [76]	Oil	Leaves	DD	No
*Nigella sativa* (Ranunculaceae)	Goat, sheep [83,84]	Oil	Seeds	DD	NE
*Opuntia ficus-indica* (Cactaceae)	Sheep [46,47]	Acetone, oil	Cladodes, seeds	DD	NE
*Origanum vulgare* (Lamiaceae)	Cattle, human, rabbit [57,58,59]	Ethanol, oil	Whole plant, leaves	DD	NE
*Phoenix dactylifera* (Arecaceae)	Buffalo, cattle, horse [40,41,42]	Semen extender	Pollen	DD	NE
*Portulaca oleracea* (Portulacaceae)	Goat [82]	Ethanol, methanol, water	Whole plant	DD	NE
*Psidium guajava* (Myrtaceae)	Goat [77]	Several steps	Leaves	DD	NE
*Punica granatum* (Lythraceae)	Buffalo, cattle, sheep [69,70,71]	Juice	Fruit	DD	NE
*Rhodiola rosea* (Crassulaceae)	Pig [49]	Water	Caudex	No	NE
*Salvia miltiorrhiza* (Lamiaceae)	Pig [60]	Several steps	Not specified	No	NE
*Salvia officinalis* (Lamiaceae)	Pig [61]	Water	Leaves	No	NE
*Salvia rosmarinus* (Lamiaceae)	Chicken, goat, pig, sheep [62,63,64,65,66,67]	Water, oil	Leaves, stems	DD	Yes
*Sambucus nigra* (Adoxaceae)	Cattle [36]	Ethanol	Fruit	DD	NE
*Schisandra chinensis* (Schisandraceae)	Cattle [87]	Ethanol	Fruit	No	Yes
*Syzygium aromaticum* (Myrtaceae)	Sheep [78]	Ethanol	Buds	DD	NE
*Syzygium cumini* (Myrtaceae)	Cattle [79]	Juice	Fruit	No	NE
*Thymus capitatus* (Lamiaceae)	Pig [63]	Oil	Not specified	Toxic	NE
*Thymus munbyanus* (Lamiaceae)	Human [68]	Oil	Leaves, flowers	Toxic	NE
*Tribulus terrestris* (Zygophyllaceae)	Human [96]	Water	Not specified	No	NE
*Ugni molinae* (Myrtaceae)	Pig [80]	Water	Fruit	DD	NE
*Urtica dioica* (Urticaceae)	Cattle [42]	Water	Not specified	No	NE
*Viscum album* (Santalaceae)	Rabbit [85]	Water	Not specified	DD	NE
*Zingiber officinale* (Zingiberaceae)	Sheep, shrimp [45,72]	Water, ethanol	Rhizome	No	Yes

DD: Dose-dependent; NE: Not evaluated.

## 4. Conclusions

Plant extracts represent a good alternative to the most common antioxidants conventionally used for sperm preservation. Among these traditional antioxidants, many of them are isolated from plants (e.g., epicatechin gallate, quercetin, aspalathin), as their inclusion in the semen extender is not profitable because of their high price. Moreover, these compounds are normally employed individually, while the use of plant extracts, as a whole, can provide a large set of secondary metabolites (e.g., phenols, terpenoids, etc.) with a powerful antioxidant effect and antimicrobial properties [97,98]. Recent advances in phytotherapy research have brought promising applications related to drug delivery. For instance, plant cells cultured in vitro release extracellular vesicles (EVs) that can be easily harvested for pharmaceutical practice [99]. Considering that some plant EVs have shown inhibitory effects against pathogenic microorganisms [99], they may be studied as possible substitutes for antibiotics in the semen preservation process. Recently, several studies reported that some polyphenols can modulate cellular metabolic pathways and transform some compounds into others, exerting not only potent antioxidant effects but also a cascade of secondary protective effects [100,101]. Some polyphenols have been shown to modulate the sperm metabolism by increasing the efficiency of mitochondrial respiration [100]. However, the source of the raw plant material, the extraction method, and the concentration of the plant extract play an important role in its effects on the sperm cell. Thus, there are many factors that affect the active constituents of plants, such as the plant age, environment (temperature, altitude, soil, rainfall, etc.), collection, processing, and storage. The different extraction methods also influence the phytochemical profile of the extract, even if using the same amount and source of plant. Moreover, the variety of plant types available on the market (e.g., fermented or unfermented, leaves or buds, etc.) exert different effects on sperm cells [53,93] because, for example, plant fermentation induces quantitative changes in the phenolic composition [102]. The part of the plant (e.g., root, bark, stem, leaf, flower, pollen, fruit) used to make the extracts is another factor to bear in mind because it may affect the chemical composition of the extract and thus its possible effects on sperm function. The selection of the plant extract concentration to be used in the sperm samples is an important step and is also influenced by the extraction method employed. Thus, in the view of the data presented in the present work, the same plant species can exert negative or positive effects on sperm parameters, in the same animal species, depending on the extract concentration employed. It is also important to bear in mind that the same concentration of plant extract can have different effects on sperm parameters depending on the preservation method used (e.g., refrigeration vs. cryopreservation). For instance, at the same concentration, *M. oleifera* extract increases shrimp sperm survival during refrigeration but not after freezing and thawing [72,73]. This could be due to the different stresses that each preservation method exerts on spermatozoa. The cryopreservation process is considered the most harmful preservation method because of the additional damages (e.g., mechanical damage) caused to the sperm’s structure. These mechanical damages might not be prevented by the action of some plant extracts. However, the addition of some plant extracts (e.g., *A. vera*) can be more appropriate for the cryopreservation process, as substitutes of cryoprotectants such as egg yolk [43], than for the refrigeration of sperm samples. Due to all the aforementioned factors, it is important to standardize the production process of plants intended for use as phytopharmaceuticals [103] and the working protocols in andrology laboratories. Moreover, it is advisable to increase the production of plant breeding lines for use as phytopharmaceuticals, where the product’s traceability is ensured (e.g., abiotic factors, nonuse of pesticides). Under such controlled conditions, the chemical profile of the extract could be more stable, thereby allowing standardization of the suitable concentrations to be used for sperm preservation. Additional studies that also involve fertility trials are desirable to further support the extensive incorporation of these natural compounds into sperm diluents. Even though the use of plant extracts as preservatives for semen storage is in its infancy, their use is promising and they can be considered natural and profitable alternatives to the most common additives currently used for sperm preservation.
